# Delivery of a mucin domain enriched in cysteine residues strengthens the intestinal mucous barrier

**DOI:** 10.1038/srep09577

**Published:** 2015-05-14

**Authors:** Valérie Gouyer, Laurent Dubuquoy, Catherine Robbe-Masselot, Christel Neut, Elisabeth Singer, Ségolène Plet, Karel Geboes, Pierre Desreumaux, Frédéric Gottrand, Jean-Luc Desseyn

**Affiliations:** 1 LIRIC – UMR 995 Inserm; Université de Lille, Lille, France; 2 CHRU de Lille, Lille, France; 3 CNRS, UMR 8576; Université de Lille, Villeneuve d′Ascq, France; 4 Department of Pathology, UZ Gent, De Pintelaan, 185, 9000 Gent, Belgium

## Abstract

A weakening of the gut mucous barrier permits an increase in the access of intestinal luminal contents to the epithelial cells, which will trigger the inflammatory response. In inflammatory bowel diseases, there is an inappropriate and ongoing activation of the immune system, possibly because the intestinal mucus is less protective against the endogenous microflora. General strategies aimed at improving the protection of the intestinal epithelium are still missing. We generated a transgenic mouse that secreted a molecule consisting of 12 consecutive copies of a mucin domain into its intestinal mucus, which is believed to modify the mucus layer by establishing reversible interactions. We showed that the mucus gel was more robust and that mucin *O*-glycosylation was altered. Notably, the gut epithelium of transgenic mice housed a greater abundance of beneficial *Lactobacillus* spp. These modifications were associated with a reduced susceptibility of transgenic mice to chemically induced colitis. Furthermore, transgenic mice cleared faster *Citrobacter rodentium* bacteria which were orally given and mice were more protected against bacterial translocation induced by gavage with adherent–invasive *Escherichia coli*. Our data show that delivering the mucin CYS domain into the gut lumen strengthens the intestinal mucus blanket which is impaired in inflammatory bowel diseases.

Millions of people worldwide suffer from intestinal infection, which continues to be a major cause of morbidity and mortality, with a higher vulnerability among children, elderly people, and individuals who are at particular risk. The entry of pathogens into the intestinal epithelial cells, which leads to inflammation, is key to a successful invasive process. These agents must cross the mucus blanket, which is the first barrier that they encounter in the intestine. A weakening of the mucous barrier permits an increase in the access of intestinal luminal contents to the epithelial cells^1^,^2^,^3^,^4^. Ulcerative colitis and Crohn's disease are the two most important inflammatory bowel diseases (IBDs), which are characterized by chronic inflammation and morphological alterations of the gut. Accumulating evidence suggests that the luminal flora is a requisite and perhaps a central actor in the development of IBDs (for review, see for example Manichanh *et al.* and Hajishengallis *at al*.^5^,^6^). The thickness of the mucus layer and its spread are decreased with increasing severity of the inflammation in IBDs^7^,^8^. In this case, the intestinal mucus becomes less protective against the endogenous microflora, which results in increased bacterial contact with the epithelium^2^.

There are five known human gel-forming mucins, which are termed MUC6, -2, -5AC, -5B, and -19. They are conserved among species^9^ and represent the major organic components in mucus^10^. MUC2 is the main secreted mucin in the intestine and, after contact with water, it forms an inner and an outer layer made of alternating laminated layers and loose curl-like structures^11^. MUC2 forms polymers and trimers that are responsible for the matrix of gels^12^. The central region of MUC2 is made of peptidic sequences that are enriched in Ser/Thr/Pro residues that carry the bulk of the *O*-glycosylation chains that are linked to the hydroxyl groups of Ser or Thr via *N*-acetylgalactosamine. This primary sugar is extended by the sequential addition of monosaccharides, which gives rise to structures that are usually terminated with the negatively charged sugar sialic acid or other residues (fucose, galactose, *N*-acetyl glucosamine, and galactosamine), which may be sulfated^13^. MUC2 is secreted as different glycoforms by glycosylation-specific goblet cell subtypes^11^ to form an inner/adherent gel that is almost devoid of bacteria and an outer/loose gel that houses bacteria^14^,^15^. Two, seven, and nine “naked” CYS domains in MUC2 (human and mouse), -5B and -5AC, respectively, interrupt or are linked to the central part of the mucin that carries extended *O*-glycosylated chains. Mucins with a higher number of CYS domains (up to 25) have been found in many species. This domain, which is characterized by 10 invariant Cys residues in higher organisms, is the best-conserved domain in secreted mucins and evolved in a concerted manner^16^. It has been suggested that hydrophobic CYS domains may establish noncovalent interactions that lead to a more complex net^17^,^18^,^19^,^20^,^21^,^22^,^23^. Consequently, adjacent CYS domains should act as natural crosslinkers of the gel that is responsible, in part, for the tight or loose net properties of mucus, according to the number of CYS domains and to the distance between adjacent CYS domains. We hypothesized that the delivery of a string of CYS domains, which are naturally found in two copies in both the human and mouse Muc2 mucin, should reinforce the mucus barrier.

## Results

### Generation of transgenic (Tg) mice and expression of the transgene

**Figure 1:**
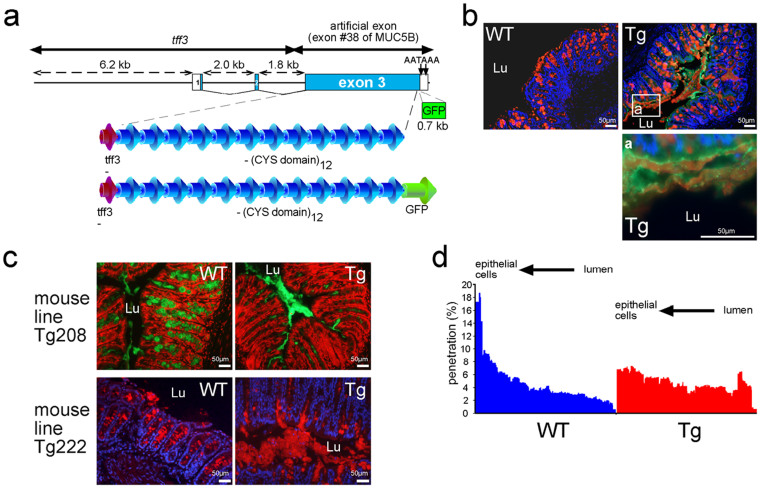
The transgene is secreted into intestinal mucus. (a). Schematic map of the two DNA fragments that were developed and employed for transgenic (Tg) mice generation and deduced peptide organization. The three exons (exons 1, 2 and 3) are indicated by boxes and numbered. White boxes represent the 5′ and 3′UTR regions. (b). Representative immunohistochemistry of colonic tissue sections of wild-type (WT) and Tg mice. The Tg product is visualized in green and Muc2 is visualized in red using the UEA1 lectin. Cells were counterstained with Hoechst 33258 (blue). (c). Representative IHC of colonic tissue sections of the two Tg mouse lines. Cells were counterstained either with propidium iodide in red (Tg208) or with Hoechst 33258 in blue (Tg222). Muc2 is visualized using an anti-Muc2 antibody in green (Tg208) or in red (Tg222). The colonic mucus was conserved in the two Tg mouse lines. Lu, lumen. (d). The distribution (%) of fluorescent beads loaded at the mucus surface into the colonic mucus in wild-type (WT; *n* = 12) and Tg (*n* = 11) mice was studied by confocal microscopy, which showed that the Tg mucus was less penetrable.

To investigate the *in vivo* role of the mucin CYS domain, we generated three Tg founders that secreted into the intestinal lumen a string of 12 consecutive CYS domains. We chose the trefoil factor 3 (*Tff3*) promoter, as TFF3 is cosecreted with MUC2 by goblet cells of the intestine. One founder was obtained using a construct without the green fluorescent protein (GFP) tag (line Tg208) and two founders were obtained using a construct with the GFP tag (line Tg222; Fig. 1a). All Tg pups developed healthy. Tg founder mice and their progeny were fertile, and preliminary data showed that the offspring of the three founders expressed the transgenes. Four wild-type (WT) and four Tg mice were sacrificed at 8 months of age, and none of the mice exhibited macroscopic tumors or any sign of intestinal inflammation. Almost all results presented here were obtained using the Tg222 line, as the GFP tag was very convenient to visualize directly transgene expression, which greatly facilitated the genotyping of our animals.

The expression of the transgene was investigated by western blotting, immunohistochemistry (IHC), and fluorescence microscopy on fresh tissues. To analyze the secretion of the transgene into the mucus gel of the colon, we performed double staining for GFP and Muc2 using the *Ulex europaeus* agglutinin 1 (UEA1) lectin. The transgene, which was expressed in goblet cells, appeared to be widespread within the Muc2 gel layers at the cell surface of the colonic epithelium, as shown by IHC (Fig. 1b & c). Western blotting using an antibody directed against the CYS domain showed that the Tg222 recombinant molecule was secreted in the colon with a MW of ~170 kDa, which was in agreement with the expected size of the Tg product (Suppl. Fig. 1a). The profile of the expression of the transgene was then analyzed by IHC and fluorescence microscopy. Its tissue expression was similar to that of the *Tff3* gene^24^, with a high expression detected in salivary glands, intestine (goblet cells), and gallbladder (Suppl. Fig. 1b). The expression of the transgene was easily observed in non-fixed colon (Suppl. Fig. 1c), ileum, and gallbladder (data not shown) under epifluorescence microscope. Transgene expression was also observed in the colon using fiber-optic endomicroscopy on anesthetized mice and on fresh ex-vivo gallbladders (Suppl. Movies 1 & 2), as well as in the ileum (data not shown). These data indicate that our Tg lines secreted a recombinant molecule consisting of 12 copies of the MUC5B CYS domain #4 into the lumen of the intestine and that the Tg molecule became tangled within the Muc2 mesh of the mucus gel.

### The mucus gel, Muc2, and goblet cells are modified

The main goal of this study was to modify the mucus layer to reinforce the mucus barrier. We began our analysis by examining more closely Muc2 expression via IHC. The mucus layer was thicker and/or more easily preserved in the colon of the two Tg lines (Tg208 and Tg222) than it was in WT mice, in which the mucus gel was barely visible (Fig. 1c). This suggests that the mucus is likely less friable after expression of the transgene. We then assessed whether the mucus properties differed between WT and Tg mice. Microspheres with the size of bacteria were added to the apical surface of colonic explants. After 45 min of sedimentation into the mucus, their distribution was studied. The relative proportion of beads was higher close to the epithelium in WT mice compared with Tg mice (Fig. 1d), which supported the notion that the transgene product is associated with a lower penetrability of the mucus blanket.

**Figure 2:**
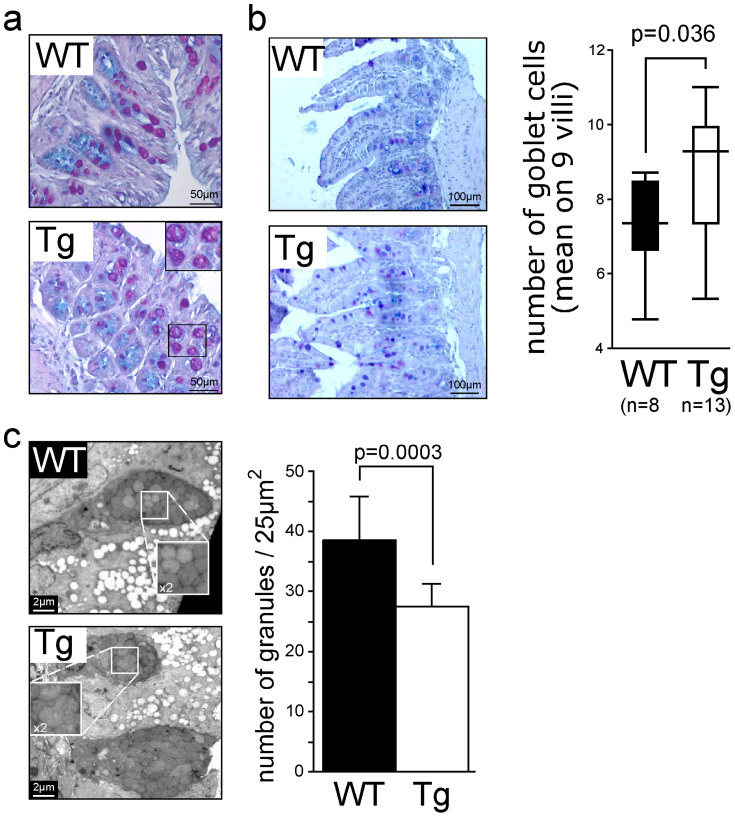
Modifications of goblet cells. (a). Some mucus granules were more vacuolated in the colon of transgenic (Tg) mice. (b). The ileum of Tg mice harbored a higher number of goblet cells, as counted on AB-PAS tissue sections. (c). Electron microscopic analysis showing that the colon of transgenic (Tg) mice harbored goblet cells with the same average surface, but with fewer mucus granules compared with wild-type (WT) mice. Close examination showed that some mucus granules were merged.

In IHC experiments, we always observed a delocalization of the Muc2 immunostaining from the goblet cells in WT mice to the conserved mucus gel layers in Tg mice. The difference of immunostaining between WT and Tg mice with the Muc2 antibody may be explained by the different forms of glycosylation of Muc2 or interaction of Muc2 with the transgene product, masking the Muc2 antigenic epitopes. To assess the expression of mucin genes, semiquantitative polymerase chain reaction (PCR; TaqMan) was performed using colonic cDNA. We did not find any significant differences in *Muc2* or *Muc6* expression levels between Tg and WT mice (Suppl. Fig. 2a); *Muc6* is weakly expressed in the human terminal ileum and right colon^25^ and in the mouse duodenum and ileum^26^. To show the specificity of mucin CYS domains for the modification of secreted mucins, we determined the relative expression levels of *Muc1*, *Muc3* (ortholog of the human *MUC17* gene^27^,^28^) and *Muc4* transcripts, the three main membrane-bound mucins expressed in colon, and no difference was found between Tg and WT mice (Suppl. Fig. 2b). Goblet cells were still present and filled with Alcian blue–periodic acid Schiff (AB–PAS) material (Fig. 2a). No obvious difference in the number of goblet cells in the colon was detected between Tg and WT mice; however, there was a significant increase in the number of goblet cells in the ileum of Tg mice (Fig. 2b). Furthermore, vacuolization of some mucus granules was sometimes observed in the colon of both the Tg208 and Tg222 mouse lines, whereas we did not observe such features in WT mice (Fig. 2a). Electron microscopy analysis revealed that the Tg mice harbored goblet cells with the same average area as those from WT mice, but with fewer mucus granules (*P* = 0.0003; Fig. 2c), because some mucus granules were merged, as illustrated in Fig. 2c.

### The gut barrier epithelium of Tg mice is not modified

To examine whether tight junctions, which play major roles as paracellular barriers, were perturbed by the expression of the transgene, we used IHC to study the two major transmembrane proteins, claudin-7 and occludin. We did not find any obvious modification in their expression patterns between the two genotypes in both the colon (Suppl. Fig. 3a) and ileum (data not shown). Next, the *in vivo* intestinal gut barrier integrity was assessed by permeability to 4 kDa fluorescein isothiocyanate (FITC)-dextran. We did not observe any differences between WT mice and Tg mice (Suppl. Fig. 3b). We concluded that the expression of the transgene did not impair gut permeability.

### Different mucin *O*-glycosylation profiles in the gut epithelium between Tg and WT mice

**Figure 3:**
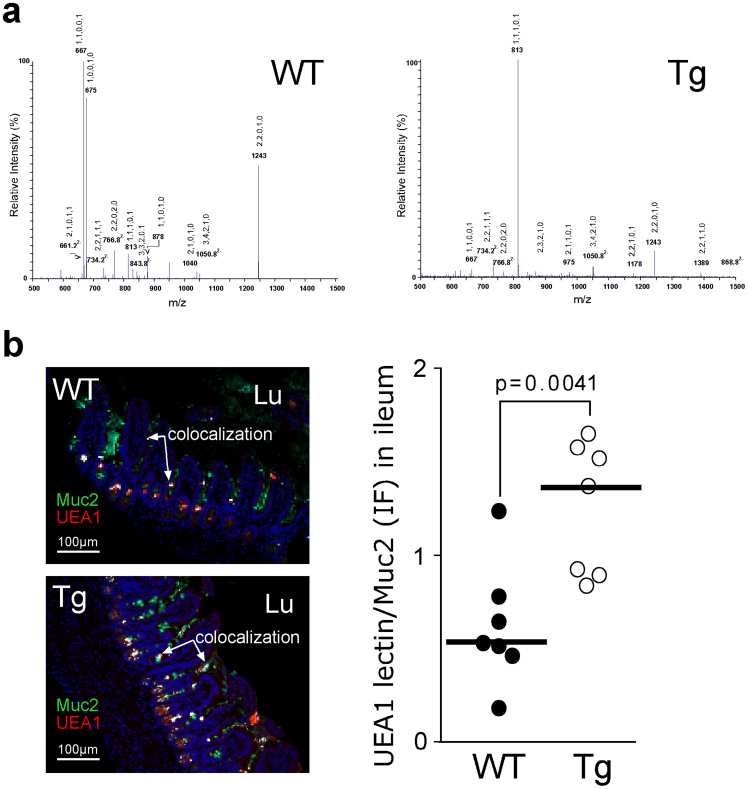
Modification of ileal mucin glycosylation. (a). Nano-electrospray-mass spectrometry of oligosaccharides from wild-type (WT) and transgenic (Tg) ileal mucins acquired in the negative ion mode. The oligosaccharide compositions of major peaks are indicated as 5 numbers separated by comma for Hex, HexNAc, Fuc, NeuAc, and SO_3_, respectively. (b). Colocalization (in white) of Muc2 (in green) and UEA1 (in red) is shown and is expressed as the ratio of UEA1/Muc2 fluorescence. Cells were counterstained with Hoechst 33258 (blue).

Colocalization study of Muc2 and the three lectins, *Sambucus nigra* (SNA), UEA1 and *Maackia amurensis* agglutinin (MAA), on gut sections was performed. This experiment confirmed the presence of a thicker mucus layer or an more preserved colonic mucus blanket in Tg mice compared with WT mice (Suppl. Fig. 4a), and showed an increased staining for the MAA lectin, which reflected a higher content of terminal sialic acid residues (α2–3) in Muc2 from Tg mice but no difference concerning the SNA lectin (data not shown). Interestingly, in the ileum, Muc2 from Tg mice contained more fucose residues than did WT mice, as shown by the analysis of Muc2 by mass spectrometry (MS, Fig. 3a). This was confirmed by the increased colocalization of the UEA1 lectin with Muc2 on ileum sections of Tg mice (Fig. 3b), and by the analysis of the carbohydrate composition of purified mucin that was isolated from mucus scraped from the ileal and colonic mucosa. In fact, the level of fucose residues was increased profoundly in the ileal mucin of Tg mice (2.3 residues of fucose per GalNac residue vs. 0.6 in WT mice; Table 1), whereas the sialic acid content was decreased in Tg mice. A decrease in the level of sialic acid and fucose residues was detected in the colonic mucins of Tg mice compared with WT mice. Moreover, the MS analysis of mucin revealed that Tg mice harbored a higher diversity of glycan chains in both the ileum (Fig. 3a) and the colon (Suppl. Fig. 4b). Quantification of the proportions of neutral/sialylated and sulfated oligosaccharides was assessed in intestinal mucus of WT and Tg mice by studying the profile of mucin *O*-glycosylation (data not shown). Whereas no differences were observed in the relative proportion of neutral glycans, an increased expression of sialylated glycans (from 17% in WT to 23% of total glycans in Tg mice) was correlated to a decreased expression of sulfated oligosaccharides (13% in Tg instead of 18.5% in WT) in colon of Tg mice, in comparison to WT mice. Ileal mucins of Tg mice were characterized by an increased in sulfate residues that was correlated to a decrease in proportion of neutral and sialylated glycans. These results showed that the *O*-glycosylation of mucins, and especially Muc2, differs greatly between Tg and WT mice.

### Tg mice harbor a greater abundance of *Lactobacillus* spp

**Figure 4:**
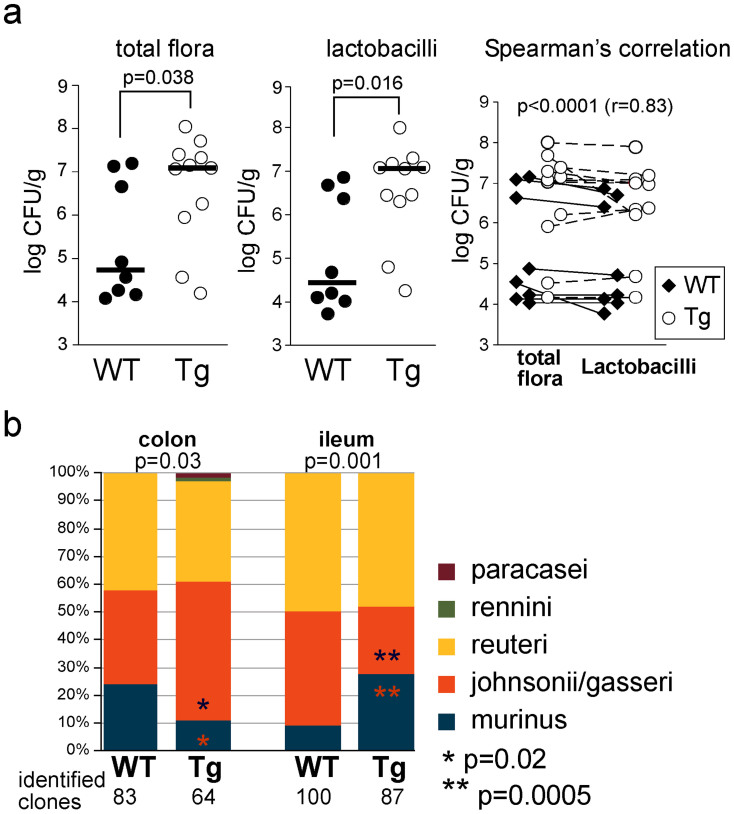
The microbiota differs between WT and Tg mice. (a). The total cultivable flora of the ileum from wild-type (WT; *n* = 8) and transgenic (Tg, *n* = 11) mice showed a higher load of Lactobacilli for Tg mice. (b). Identification of *Lactobacillus* ssp. by MS showed an alteration of the *Lactobacillus* composition in both the colon and ileum between the two genotypes.

We studied the gut microbiota because the intestinal bacteria drive, in part, mucin *O*-glycosylation^29^,^30^. We analyzed the total cultivable flora of brother–sister WT and Tg mice housed in the same cage. We found that Tg mice harbored a significantly greater abundance of probiotic *Lactobacillus* spp. (2.5 log) in the ileum compared with their WT littermates (Fig. 4a). No difference was found between the two genotypes, probably because of the high content of bacteria in the colon. The identification of *Lactobacillus* spp. was then performed by MS, a fast and cost-effective identification method, which showed an alteration of the *Lactobacillus* composition in both the colon and ileum (*P* = 0.03 and *P* = 0.001, respectively) between WT and Tg mice (Fig. 4b). The relative abundance of *L. johnsonii/gasseri* and *L. murinus* differed between the colon and the ileum of Tg mice. These findings suggest that the mucous barrier could be strengthened in part by the increased number of lactobacilli, which are beneficial to the host. We then decided to test this hypothesis by challenging mice with a toxic chemical and noncommensal bacteria.

### Tg mice are less susceptible to chemically induced colitis

**Figure 5:**
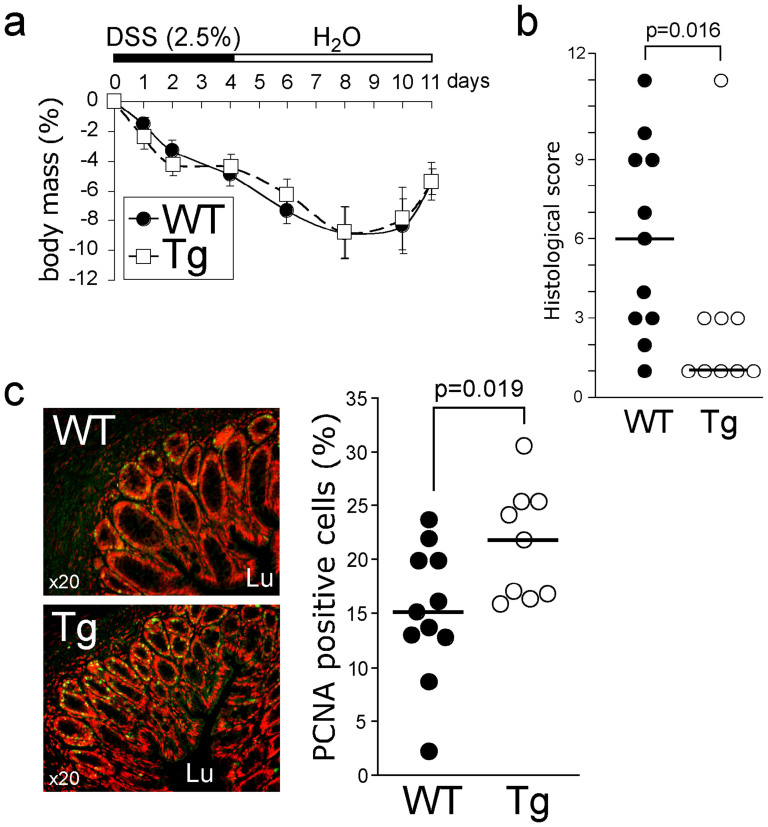
The transgene protects against dextran sodium sulfate (DSS)-induced colitis. (a). Wild-type (WT; *n* = 11) and transgenic (Tg, *n* = 9) mice were treated with 2.5% DSS or phosphate-buffered saline (PBS) for 5 days, and then received water for 7 days. Body mass was scored daily. (b). Histological score^76^ was calculated at day 12. (c). Proliferating cell nuclear antigen (PCNA) immunostaining of colon sections and its quantification showed an increase in epithelial cell proliferation in Tg mice.

It has been reported that Tff3 is upregulated after experimental colitis induced by intrarectal administration of acetic acid^31^. Because the transgene was driven by the Tff3 promoter, we investigate whether Tg mice are less susceptible to chemically induced colitis. We challenged WT and Tg mice with dextran sodium sulfate (DSS) for 5 days, followed by 7 days of water administration, to allow the repair of the epithelium^32^. No difference was observed regarding body mass variation between the two genotypes (Fig. 5a). No difference in terms of cytokine expression (IL1b, IL6, IL17α, TNFα, and INFγ) was found at the mRNA level, and myeloperoxidase (MPO) activity was similar between the two groups (data not shown). However, Tg mice had a better histological score (*P* = 0.016, Fig. 5b). Criteria accounting for this better score (Suppl. Table 2) showed that the transgene expression induced faster tissue regeneration (*P* = 0.025) and lower severity score (*P* = 0.022). To confirm the role of the transgene on wound healing, we used IHC to quantify epithelial cell proliferation in the colon of animals from the two groups. We found a higher proliferative index in Tg mice (*P* = 0.019; Fig. 5c). The faster regeneration of the epithelium observed after chemical injury suggests that Tg mice are less susceptible to chemically induced colitis compared with WT mice.

### The transgene protects mice against *Citrobacter rodentium* colonization

**Figure 6:**
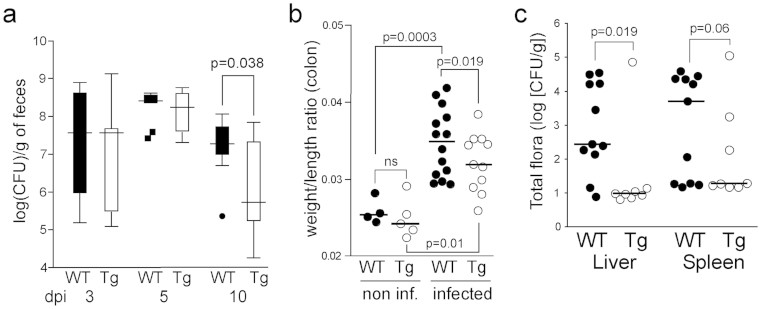
The transgene protects against bacterial challenges. (a). Wild-type (WT; *n* = 14) and transgenic (Tg, *n* = 11) mice were infected by oral gavage with 100 μL of an overnight culture of *C. rodentium* (~3.6 × 10^9^ CFU) and sacrificed at 10 dpi. Feces were collected from live mice at 3, 5, and 10 dpi and *C. rodentium* was counted. (b). The colon weight/length ratio was calculated at 10 dpi. (c). WT (11) and Tg (8) mice were infected by oral gavage with 100 μL of an overnight culture of the clinical AIEC strain 06362^33^ containing ~1 × 10^9^ CFU bacteria on days 1 and 2, and were sacrificed on day 7. Liver and spleen homogenates were serially diluted in PBS and plated to determine the translocation of the total cultivable bacteria.

To test whether transgenic mice are less susceptible to pathogen colonization, Tg and control WT mice were challenged orally with *Citrobacter rodentium*, a natural murine A/E pathogen related to diarrheagenic A/E *Escherichia coli*. Fecal *C. rodentium* load was quantified at 3, 5, and 10 days postinoculation (dpi), and a peak at day 5 and a decrease of 1.5 log in the pathogen load were detected at 10 dpi in the Tg group (*P* = 0.038; Fig. 6a). We did not observe any differences between the two genotypes regarding relative variation of body mass, pathological lesions, and bacterial translocation (data not shown). Importantly, infected Tg mice had a lower colonic weight/length ratio than did infected WT mice at 10 dpi (Fig. 6b). The thinner colon wall and smaller shrinkage detected in Tg mice after infection are in agreement with the faster clearance of *C. rodentium* in fecal stools of Tg mice.

### The transgene protects mice against bacterial translocation

Next, we administrated orally the adherent–invasive *E. coli* (AIEC) strain 06362^33^ isolated from patients with Crohn's disease. AIEC is a human-specific *E. coli* that is found with a high prevalence in the intestine of patients with Crohn's disease (36.4% in patients vs 6.2% in the normal population)^34^,^35^. The clinical AIEC strain was administered intragastrically, and the translocation of total cultivable bacteria in the liver and spleen was measured at 7 dpi. Because AIEC is not pathogenic in rodents, no mice died during the experimental period. Bacterial translocation was lower in the liver of animals in the Tg group than it was in that of animals in the WT group (Fig. 6c; 2.5 log vs. 1 log, respectively), and the bacterial load was slightly lower in the spleen of animals in the Tg group (*P* = 0.06).

## Discussion

The intestinal MUC2 gel-forming mucin prevents the adhesion of pathogens to the gut epithelium^3^,^14^,^36^,^37^. It is believed that CYS domains are involved in the hydrophobic interactions between mucins. Such interactions are probably crucial to the barrier properties of mucin hydrogels^17^,^18^,^20^, but direct proof of this function is missing. The objective of the proposed research was to test whether the delivery of a molecule made of mucin CYS domains into the mouse gut could modify the mucus layer and strengthen the mucous barrier. Although CYS domains are interspersed with *O*-glycosylated regions in the human/rodent MUC2/Muc2 mucin, a cluster of CYS domains is found close to *O*-glycosylated regions, at least in MUC5AC and MUC5B^38^. Furthermore, molecules consisting only of CYS domains exist and may represent new compounds that can be used to reinforce the mucus blanket. This is the case of Oikosin1, which is a natural molecule consisting of consecutive CYS domains without mucin *O*-glycosylated regions^39^. This molecule, which contains 13 CYS domains, is cosecreted with mucins by the small marine organism *Oikopleura dioica* and participates in the formation of the mucus house, which filters nutrients. We successfully constructed two large plasmid vectors (20.2 and 18.9 for Tg208 and Tg222, respectively) to deliver a minigene encoding 12 consecutive CYS domains. The Tg product was cosecreted with Muc2 and as interspersed within the intestinal mucus layers. Because of the high water content of mucus, tissues must be fixed in Carnoy's solution in order to provide the most satisfactory preservation of the mucus layers in paraffin blocks^11^,^40^,^41^. Remarkably, mucus layers of transgenic mice were easily preserved in paraformaldehyde fixative as illustrated for example in Fig. 1b and 1c. This result indicates that the mucus was modified in Tg mice. Consequently, the mucus blanket appeared much thicker in Tg mice as well as less penetrable by inert fluorescent particles. Tg mice were healthy and several modifications were detected regarding the intestinal gut barrier: granules of goblet cells, *O*-glycosylation of the intestinal gel-forming mucin, and, more importantly, alteration of the intestinal microbiota, with a significant increase in the abundance of probiotic lactobacilli.

A similar morphological modification of the mucus layer has been observed in the colon of Tat element modulatory factor (TMF)-deficient mice^42^. TMF is a Golgi-associated protein that accumulates in colonic enterocytes and goblet cells. The gut epithelium of TMF^–/–^ mice harbored a thicker mucus layer, and mucus granules did not fuse during the secretion process, as shown for WT mice. In our case, the Tg mouse strain showed some granule fusion before secretion. Perhaps the glycosylation modification of Muc2 or the interaction between the transgene product and Muc2 modifies vesicles tethering in goblet cells. The mucus gel of the Tg mice appeared thicker and likely more robust than was that of the control WT littermates, as shown by the preservation of the mucus for histological analysis, similar to that observed in TMF^–/–^ mice^42^. However, to our surprise, and in contrast with TMF^–/–^ mice, our Tg mice did not exhibit an increase in the Muc2 mRNA level, although an increase in the number of goblet cells was noticed. Interestingly, TMF^–/–^ mice host a significantly distinct bacterial population compared with WT mice, with a higher relative abundance of the *Lactobacillus* group^43^, which is associated with decreased susceptibility to IBDs^44^.

We found a direct relationship between the much higher abundance of the total intestinal cultivable bacterial community observed in Tg mice compared with WT brother–sister mice housed in the same cage and a much higher abundance of *Lactobacillus* spp. Commensal bacteria are crucial for building the intestinal barrier and defending against pathogens that need to compete with the host microbiota^45^. The results of the present study strongly suggest the presence of a relationship between mucin *O*-glycosylation and the abundance of lactobacilli. Because the interaction between the microflora and the host drives, in part, the maturation of the glycosylation of the host^46^,^47^, it is possible that the *O*-glycosylation modifications observed in the gut of Tg mice were caused by the alteration of the *Lactobacillus* flora. The most striking glycosylation alteration was the higher abundance of fucosylated glycotopes in the intestinal mucin of Tg mice. Fucosylated glycoconjugates are important as a source of nutriments for the microflora^48^ and may also be important for host-microbe interactions^49^. It is not known if *Lactobacillus* spp. induce mucin fucosylation, as shown for *Bacteroides thetaiotaomicron*, which is a model component of the intestinal microbiota^29^. We may also expect that the Tg product, which was cosecreted with the main intestinal mucin and impacted goblet cell morphology, affects the mucin *O*-glycosylation in a bacteria-independent manner. One hypothesis to explain this result is that the production and maturation of the transgene product slow down the maturation process of Muc2. Axenic mice carrying our transgene or culture of mucous cells expressing the transgene should be helpful to determine whether mucin *O*-glycosylation is modified by the transgene and/or lactobacilli.

The four main *Lactobacillus* ssp. identified here in the mouse gut epithelium, *L. reuteri*, *L. johnsonii/gasseri*, and *L. murinus*, have been shown to affect human health beneficially^50^,^51^,^52^,^53^. *Lactobacillus* ssp. colonization of the mucus gut is necessary to maintain the defense at the mucosal surface by preventing colonization by pathogens, and requires an interaction between *Lactobacillus* ssp. and mucus^54^ that seems to rely mainly on the terminal mucin glycans^55^. Among the monosaccharides of mucins, fucose, which was abundant in the intestinal mucin of Tg mice, has been reported to be the most important factor for the interaction between the commensal microflora and the host. More specifically, *Lactobacillus* binds di- and trisaccharide carbohydrate antigens A/B/H, which all contain a fucose residue^56^,^57^. Furthermore, fucose inhibits the adhesion between mucus and a mucus-binding protein of *L. reuteri*, which is conserved in *Lactobacillus* ssp. of the gastrointestinal tract^55^,^58^. These data suggest that the higher fucosylation of intestinal mucin observed after the delivery of the mucin CYS domain contributes to the reinforcement of the gut barrier.

Mice expressing the CYS transgene were less susceptible to DSS-induced colitis and were less susceptible to colonization with the rodent pathogen *C. rodentium*. These beneficial effects are likely linked to a more robust mucus, but may also stem from the abundance of *Lactobacillus* spp., which protects mice against experimental colitis^43^,^59^ and diminishes the severity of *C. rodentium* experimental infection^60^. Admittedly, an imbalance in bacterial composition may be the main cause of mucosal inflammation in IBDs. Because clinical studies have suggested that the adherent–invasive *E. coli* (AIEC) pathovar may be linked to IBDs^34^,^35^, we challenged mice with an AIEC clone which is more virulent than the LF82 pathovar^33^. Although AIEC is a nonpathogenic organism when administered orally to mice, AIEC gavage induced a translocation of gut bacteria to tissues that are normally sterile, with a clear decrease in susceptibility observed in mice that carried the transgene. Modifications of both the mucus characteristics and of the microbiota may explain the finding that Tg mice were more protected against bacterial translocation. This is, in part, supported by the fact that lactobacilli inhibit the interaction between AIEC and intestinal epithelial cells^61^,^62^.

Collectively, we described here a clearly positive relationship between the delivery of a molecule consisting of 12 consecutive CYS domains from mucin into the gut lumen and modifications of the mucus layer, with a reinforcement of the defense and protection of the gut epithelium. The introduction of the mucin CYS domain into the intestinal lumen might represent a promising noninvasive strategy against intestinal infection and IBDs.

## Methods

### Transgene construction

Two transgenes driven by the 6.4 kb mouse intestinal trefoil factor trefoil factor-3 (*Tff3*) promoter were prepared. They contained the full-length first intron (2 kb), the 1.7 kb 5′ part of intron 2, and the two first exons of the mouse *Tff3* gene, which encode the signal peptide. This 10.3 kb insert (cloned in the pKSII plasmid vector) was a kind gift of DK Podolski^63^. A genomic fragment containing an artificial exon (see below) encoding 12 identical CYS sequences from MUC5B, followed or not by the enhanced GFP (EGFP)^64^ sequence in frame and a 3′ untranslated region (UTR) sequence, was inserted downstream of the *Tff3* genomic sequence. A schematic representation of the map of the two constructs is given in Fig. 1a. Briefly, the 18.1 kb and 18.9 kb Tg plasmid constructs were created as follows. The BEN2 cosmid clone containing the large central exon of *MUC5B*^65^ was used to amplify an intronless fragment that encodes a short peptide, followed by the CYS domain #4 with two oligonucleotides containing a *Bam*HI restriction sequence and a *Bgl*II restriction sequence, respectively. The 0.33 kb PCR product encoding the short peptide PSTPATRSTSAPITTVVTMG followed by the CYS sequence was cloned into the pCR4 vector (Invitrogen). We chose the CYS domain #4 of the human *MUC5B* mucin because we utilize specific antibodies against a peptide of this domain, i.e., the monoclonal antibodies EU-MUC5Ba and EU-MUC5Bb^66^ and the polyclonal antibody LUM5B^67^. Several rounds of excision of the *Bam*HI–*Bgl*II genomic insert, and subcloning of the insert into the *Bgl*II-opened pCR4 plasmid containing one or more CYS sequences allowed the generation of a pCR4 vector containing 12 CYS copies of the CYS sequence in frame and in the same orientation. Exon 38 (101 bp) of *MUC5*B^68^ with intronic bordering sequences (50 and 56 bp, respectively) was amplified by PCR, and the product was cloned in the pCR4 vector, to create exon 3 of the transgenes. An *Xba*I site was added in the 3′ intronic sequence during the PCR amplification. Two *Bam*HI restriction sequences were introduced into the exonic sequence using the QuikChange Site-Directed Mutagenesis protocol from Stratagene. The 56 bp *Bam*HI–*Bam*HI exonic sequence was then replaced by the 4356 bp *Bam*HI–*Bgl*II sequence encoding the 12 CYS domains in tandem. The *Tff3* nucleic acid sequence encoding the signal peptide and the sequence encoding the 12 CYS domains were in frame. The 3′UTR (0.2 kb) sequence from the early *SV40* small antigen gene was added into the artificial exon 3, downstream of the sequence encoding the 12 CYS sequences, giving rise to the Tg208 transgene. The GFP sequence (0.76 kb) that was amplified by PCR from the pEGFP-N1 plasmid (Clontech) was subcloned in frame between the last CYS sequence and the 3′UTR sequence, giving rise to the Tg222 transgene. Parts of the transgene inserted sequences were checked by nucleotide sequencing, and the gene structure was verified using the GENSCANW Web server (http://genscanw.biosino.org/).

### Generation of Tg mice and genotyping

To remove the plasmid backbone from the two constructs, the 14.9 and 18.9 kb DNA fragments comprising the *Tff3* promoter linked to *Tff3* exons 1 and 2 followed by the artificial exon encoding 12 CYS sequences in tandem and the same construct fused to the EGFP sequence, respectively, were released by digestion with *Eco*RV (for the *Tff3* promoter 5′ end) and *Sac*II (for the 3′UTR end) using standard techniques. The linearized DNA fragments were purified by agarose gel electrophoresis, followed by electroelution and conventional phenol/chloroform extraction and ethanol precipitation techniques^69^. The two DNA fragments were microinjected using standard techniques^70^. Four-week-old mice were screened for the presence of the transgene by PCR analysis using tail DNA extracted as described elsewhere^71^. The primers used were 5′–ACCTACTCCAACATCCGTGC–3′ (forward) and 5′–GTAGGTGTCAAAGTCCCCGC–3′ (reverse). The PCR conditions used for the genotyping included a denaturation step at 94°C for 2 min, followed by 35 cycles of incubation at 95°C for 30 s, 57°C for 40 s, and 72°C for 30 s. The amplified products (400 bp for the Tg mice and no amplification for WT mice) were subjected to electrophoresis on a 12% acrylamide/bisacrylamide gel. Mice were kept on a standard chow diet *ad libitum* in a specific pathogen-free environment. The Tg lines were maintained by breeding heterozygous mice with C57BL/6 mice. In all experiments, Tg mice were compared with their WT littermates. The animal procedure adopted in this study was approved by the Animal Care Ethics Committee of the Nord-Pas-de-Calais region (approval ID: CEEA182012). The animal care and all procedures were in accordance with the French Guidelines for the Care and Use of Laboratory Animals and with the guidelines of the European Union. The two Tg lines described here were registered under the same GMO number (5287) at the French Minister of Education and Research.

### Tissue collection

Mice were killed by cervical dislocation and their gallbladder, salivary glands, and intestines were resected. The intestines were divided into duodenum, ileum, proximal colon, and distal colon for further analysis. Tissues were immediately placed in 4% paraformaldehyde for 18 h or in fresh Carnoy's fixative for 4 h for histological studies, or were frozen in liquid nitrogen and stored at −80°C for subsequent analysis.

### Analysis of mucin *O*-glycosylation

Mice were killed and mucus from the colon and ileum was removed immediately by scraping the mucosa (*n* = 5 mice/group). Mucins were solubilized and purified by isopycnic density-gradient centrifugation^72^. The mucin-containing fractions were pooled, dialyzed against water, lyophilized, and further submitted to β-elimination under reductive conditions (0.1 M NaOH and 1 M KBH_4_ for 24 h at 45°C). Oligosaccharide–alditols were analyzed by nanoelectrospray mass spectrometry (MS) in positive and negative ion modes. All analyses were performed on a Q-STAR Pulsar quadrupole time-of-flight (TOF) mass spectrometer (Applied Biosystems/MDS Sciex, Toronto, Canada) fitted with a nanoelectrospray ion source (Protana, Odense, Denmark). Oligosaccharides dissolved in water (60 pmol/μL) were acidified by addition of an equal volume of methanol/0.1% formic acid and sprayed from gold-coated “medium-length” borosilicate capillaries (Protana). A potential of ±800 V was applied to the capillary tip, the focusing potential was set at ±100 V, and the declustering potential varied between ±60 V and ±110 V. For the recording of conventional mass spectra, TOF data were acquired via the accumulation of 10 multiple channel acquisition scans over mass ranges of m/z 400–2,000. In collision-induced dissociation tandem MS analyses, multiple charged ions were fragmented using nitrogen as the collision gas (5.3 × 10^−5^ Torr) and a collision energy that varied between ±40 and ±90 eV, to obtain optimal fragmentation. The collision-induced dissociation spectra were recorded on the orthogonal TOF analyzer over a range of m/z 80–2,000. Data acquisition was optimized to yield the highest possible resolution and the best signal-to*-*noise ratio, even in the case of low-abundance signals. Typically, the full width at half maximum was 7,000 in the measured mass ranges. External calibration was performed prior to each measure using a 4 pmol/μL solution of taurocholic acid in acetonitrile/water (50:50, v/v) containing 2 mM of ammonium acetate. Permethylation of the mixture of oligosaccharide alditols was carried out with the sodium hydroxyde procedure. After derivation, the reaction products were dissolved in 200 μL of methanol and further purified on a C18 Sep-Pak (Waters, Milford MA). Permethylated oligosaccharides were analyzed by MALDI-TOF mass spectrometry in positive ion reflective mode as [M+Na]+. The relative proportions of each oligosaccharide were calculated by integration of peaks on MS spectra, and are expressed as percentages of the total.

### Monosaccharide analysis

The monosaccharide composition of the mucins was determined by gas chromatography on a Shimadzu gas chromatographer equipped with a 25 × 0.32 mm CP-Sil5 CB Low-bleed/MS capillary column and a 0.25 μm film phase (Chrompack France, Les Ullis, France) after methanolysis (0.5 M HCl–methanol for 24 h at 80°C), *N*-reacetylation, and trimethylsilylation^73^.

### SDS–PAGE and western blot analyses

Protein samples were prepared according to standard procedures, mixed 1:4 with Laemmli reducing loading buffer (4×), and boiled for 5 min in a water bath. Proteins were separated on 12.5% SDS–PAGE for 2 h at 100 V. Western blot analyses were performed by electroblotting onto PVDF membranes. The membranes were blocked with 5% (w/v) skimmed milk in TBS containing 0.1% (v/v) Tween 20 (blocking solution) for 1 h, and incubated with the rabbit anti-MUC5B polyclonal antibody LUM5B-2^67^, which is directed against the MUC5B CYS domain (12 copies of which were present in the Tg product).

### Histology and immunohistochemistry

Five-micron-thick sections were prepared. AB–PAS staining and hematoxylin & eosin staining were performed as described previously^11^. To count goblet cells, the total number of PAS-positive cells was determined in 10 longitudinally sectioned crypts of the colon or villi of the ileum per section. The number of goblet cells was expressed as the total number of PAS-positive cells per crypt or per villus. IHC staining was performed as described previously^11^. Anti-MUC2, anti-proliferating cell nuclear antigen (PCNA), and anti-GFP staining was performed using the H300 anti-MUC2 polyclonal antibody (Santa Cruz), the PC10 anti-PCNA monoclonal antibody (Abcam), and the ab290 anti-GFP polyclonal antibody (Abcam), respectively. When using the PC10 and ab290 antibodies, a heat-mediated antigen retrieval step was performed before carrying out the IHC protocol, as follows: sections were immersed in sodium citrate buffer (10 mM sodium citrate, 0.05% Tween, pH 6.0) at 95–100°C for 20 min and then at room temperature for 20 min. Slides were washed in phosphate-buffered saline (PBS) and labeling was performed. The SNA lectin, which recognizes the epitope of sialic acid α-2,6-galactose, the UEA1 lectin, which recognizes H type 2 epitopes (Fuc α-1,2-Gal) and the MAA lectin, which recognizes the epitope of sialic acid α-2,3-galactose, were conjugated directly to TRITC (EY Laboratories, Biovalley, Marne La Vallee, France) and were used at 25 μg/mL. The sections used in immunofluorescence experiments were counterstained with Hoechst 33258 (1:1,000) or propidium iodide (1 ng/mL).

### Mucus penetrability

Fluorescent beads with a diameter of 1 μm (Biovalley, Marne La Vallee, France) were loaded (10 μL; ~10^6^ beads) at the surface of fresh colon samples that were opened lengthwise (12 WT and 11 Tg mice, respectively). After 45 min, particle penetration was recorded by confocal imaging in an XY stack using a Zeiss LSM 710 confocal microscope and the ZEN 2009 software (Zeiss, Germany). Images were analyzed using the ImageJ software (National Institutes of Health, Bethesda, MD). For each sample tissue, the total number of beads was normalized to 1,000 and the number of sections was normalized to 210. The particle distribution is presented as the percentage of beads/section.

### Immunofluorescence analysis

Multilabel immunofluorescence analysis was performed on a Leica DM4000B or a Zeiss LSM 710 confocal microscope. Images were acquired and minimally processed by importing them into the GNU image manipulation program GIMP and the ImageJ software. Quantification of Muc2 and UEA1 or SNA colocalization in the ileum and in the colon (*n* = 7 mice/group) was performed on 4–7 villi or crypts/mouse. Fluorescence was measured and expressed as the UEA1/Muc2 and SNA/Muc2 (data not shown) ratio relative to the area of the villus or the crypt.

### Electron microscopy

For ultrastructural analysis, colon biopsies were fixed with 1% paraformaldehyde and 2% glutaraldehyde in 0.1 M phosphate buffer (pH 7.2) for one night. Tissues were then washed in phosphate buffer, harvested, and postfixed with 1% osmium tetroxide (OsO_4_) for 2 h. They were dehydrated in a graded ethanol series and embedded in Araldite resin. Ultrathin sections (90 nm thick) were cut on an ultramicrotome (Ultracut E; Reichert-Jung, Leica, France), collected on parlodion-coated nickel grids, and stained successively with 2% uranyl acetate and lead citrate. Images were acquired using a transmission electron microscope (Zeiss EM902; Zeiss, Germany) equipped with an Orius camera interface. Pictures were taken at a resolution of 8 million pixels using the Digital Micrograph software (Gatan, France). Analysis of goblet cell morphological changes was realized by counting the total number of mucus granules within 2 μm^2^ and 5 μm^2^ boxes by two independent observers who were unaware of the genotype of the animals. Two WT and two Tg mice were used. At least 10 sections with whole and intact goblet cells were analyzed per experiment, and the results were averaged.

### *In vivo* fibered confocal laser microscopy

Mice were anesthetized using ketamine/xylazine (100 and 10 mg/kg, respectively). Fluorescence was analyzed using the Cellvizio (laser excitation wavelength, 488 nm) apparatus (Mauna Kea Technologies) with the ultra-thin flexible fibered microprobe ProFlex PF-0150.

### RT–qPCR

Total RNA extraction, cDNA synthesis, and PCR experiments using the 18S ribosomal RNA as an internal positive control were performed as described previously^74^. Primer and TaqMan probe sequences were selected using the Primer3 Output program Technology (MIT) and are listed in Suppl. Table 1. The oligonucleotides used to measure *Muc6* and *Muc4* gene expression have been published previously^26^,^74^. All samples were measured in triplicate. The cycle threshold values of all samples were measured using the ABI Prism 7700 sequence detector system (Applied Biosystems), and target mRNA expression levels were normalized to the mRNA levels of 18S ribosomal RNA. Relative amounts of target genes were calculated using the ΔΔCt method.

### FITC-dextran intestinal permeability assay

For permeability measurement *in vivo*, eight mice per group were orally inoculated with 4 kDa FITC-dextran (440 mg/kg) 4 h before they were killed. Blood was collected by cardiac puncture, and serum FITC-dextran concentrations were determined by fluorometry at 485 nm using a microplate reader (Mithras).

### Microbiological analysis

Anaerobic and aerobic cultivable microflora from fresh fecal stools and tissues were quantified as described previously^75^. Total counts were performed, and different types of colonies were subcultured and identified according to established morphological and biochemical criteria. Quantitative results are expressed in log colony-forming units (CFU) g^−1^. The threshold of detection was 10^4^ CFU g^−1^.

### *Lactobacillus* spp. identification by matrix-assisted laser desorption ionization/TOF (MALDI-TOF)

Briefly, each lactobacillus isolate was cultured from 24 to 48 h in Difco lactobacilli MRS broth (BD, France) with 5% CO_2_, and the bacterial culture was centrifuged for 5 min at 6,000 × *g*. The supernatant was then discarded and the bacteria were resuspended in 300 μL of double-distilled water to 0.5–1 McFarland. Nine hundred microliter of 100% ethanol (Sigma-Aldrich, France) was added and the bacteria were vortexed before centrifugation at top speed (14,000 × *g*) for 2 min. The supernatant was discarded, bacteria were resuspended in 10–50 μL of formic acid (Sigma-Aldrich), and the same volume of acetonitrile (Sigma-Aldrich) was added and thoroughly mixed, followed by centrifugation at top speed for 2 min. A sample of the supernatant was spotted onto a ground-steel MALDI target according to the manufacturer's instructions (Bruker Daltonics, Bremen, Germany), dried at room temperature, covered with 1.5 μL of matrix solution (10 mg/mL of α-cyano-4-hydroxycinnamic acid in acetonitrile:water:trifluoroacetic acid, 50:47.5:2.5 vol/vol/vol), and analyzed on a MALDI–TOF Biotyper instrument (Bruker Daltonics). The acquired mass spectrum was used for a search in the MALDI Biotyper database using the Bruker Biotyper 2.0 software. Lactobacilli isolates with a score ≥ 2,000 were kept for our study. Because *L. johnsonii* and *L. gasseri* often exhibited a similar score (difference < 10%), we considered all *L. johnsonii* or *L. gasseri* as the same sp. *L.* *johnsonii/gasseri*.

### Chemically induced colitis

For chemical colitis induction, mice were administered 2.5% DSS (MW, 40 kDa; TdB Consultancy, Uppsala, Sweden) dissolved in drinking water for 5 days, followed by 7 days of regular drinking water, for recovery. Fresh DSS solution was prepared daily. A histological grading of the colitis according to Dieleman *et al*.^76^ was performed by two investigators who were unaware of the genotype. The maximum score that can be obtained from this scoring system is 20. For the quantification of cells that were positive for the PCNA in the colon, the total number of epithelial cells and PCNA-positive cells were determined for 10–20 longitudinally sectioned crypts per section. The number of PCNA-positive cells was expressed as the total number of PCNA-positive cells per 100 epithelial cells.

### Myeloperoxidase assay

The activity of the MPO enzyme was determined in colon tissues according to a method adapted from Han et al.^77^. A standard curve was prepared using MPO from human neutrophils at concentrations from 0 to 0.5 U/100 μL. Absorbance was measured at 450 nm on a microplate reader (Mithras). One unit of MPO activity was defined as the quantity of MPO that was necessary to degrade 1 μmoL of hydrogen peroxide/min/mL at 25°C. The MPO activity was expressed as MPO U/g of colonic tissue.

### Bacterial challenges: *Citrobacter rodentium* challenge

Mice were infected by oral gavage with 100 μL of an overnight culture of Luria broth (LB) containing approximately 3.5 × 10^9^ CFU of the kanamycin-resistant *C. rodentium* strain DBS120^78^ and sacrificed 10 dpi. No mice died during the experimental period. For fecal bacterial burden analysis, stools were collected from live mice at various times postinoculation. To count bacteria within tissues and feces (at 3, 5, and 10 dpi), biopsy specimens of the colon, ileum, mesenteric lymph nodes, mesenteric fat, liver, spleen, and feces were collected in a preweighed 2.0 mL microtube containing 1.0 mL of PBS. Tissue and feces were weighed and then homogenized with a pellet pestle. Feces homogenates were serially diluted in PBS, plated onto LB agar plates containing 100 mg/mL of kanamycin sulfate (Sigma-Aldrich), and incubated overnight at 37°C. *C. rodentium* colonies were counted the following day and normalized to the stool weight (per gram).

To assess tissue pathology, we used a scoring system according to Bergstrom et al.^3^. The maximum score that can be obtained from this scoring system is 15. The colon wet weight/length ratio (mg/cm) was also calculated as a criterion of injury.

### Adherent–invasive Escherichia coli (AIEC) challenge

For AIEC challenge, 11 WT and eight Tg mice aged 6–9 weeks of age were infected by oral gavage with 100 μL of an overnight culture of the clinical AIEC strain 06362^33^ containing approximately 1.10^9^ CFU bacteria at days 1 and 2, and were sacrificed by cervical dislocation at day 7 for analysis. Liver and spleen homogenates were serially diluted in PBS and plated, to determine bacterial translocation as described above.

### Statistical analyses

The Wilcoxon–Mann–Whitney test was used to compare unpaired data. Pearson's chi-squared test was used to analyze categorical variables. Correlations between variables were calculated using Spearman's rank correlation tests (*r*_s_). A *P* value < 0.05 was considered significant. The data were analyzed using StatXact® 6.0 (Cytel Studio, Cambridge, MA) for exact nonparametric inference.

## Author Contributions

J.-L.D. formulated the original hypothesis and designed the construct. Animal experiments were performed by V.G., J.-L.D. and L.D. microbiology analysis was performed by C.N., S.P. and E.S. *O*-glycosylation analysis was performed by C.R.M. genotyping, histology, immunohistochemistry, and fluorescence microscopy were performed by V.G. and S.P. histological analyses were performed by K.G. and V.G. P.D. provided advice and directions to the study; J.-L.D., V.G. and F.G. wrote the manuscript; and all authors contributed to the discussion of the results and reviewed the manuscript before submission.

## Supplementary Material

Supplementary Information Supplementary Information

Supplementary Information Intrarectal endoconfocal imaging of anesthetized mice

Supplementary Information Endoconfocal imaging of the gallbladder of a transgenic mouse

## Figures and Tables

**Table 1:** Monosaccharide composition of native mucins isolated from colonic and ileal mucosa of wild-type (WT) and transgenic (Tg) mice. The molar ratio of the different monosaccharides was calculated based on one GalNAc residue

	aFuc	bGal	cGalNAc	dGlcNAc	eNeuAc
Colon WT	1.8	2.5	1.0	2.9	2.9
Colon Tg	1.4	2.4	1.0	2.5	2.4
Ileum WT	0.6	1.5	1.0	1.2	2.0
Ileum Tg	2.3	1.8	1.0	1.4	1.6

aFucose; ^b^Galactose; ^c^N-acetyl galactosamine; ^d^N-acetyl glucosamine; ^e^N-acetyl neuraminic acid. Data are representative of two independent experiments and monosaccharide composition has been determined in duplicate.
