# Supplementary material for: Delivery of a mucin domain enriched in cysteine residues strengthens the intestinal mucous barrier
Source: Sci Rep. 2015 May 14;5:9577. doi: 10.1038/srep09577 (PMC4431476; doi:10.1038/srep09577)
Supplement: Supplementary Information [file srep09577-s1.pdf]

Delivery of a mucin domain enriched in cysteine residues strengthens the intestinal mucous barrier

Valérie Gouyer, Laurent Dubuquoy, Catherine Robbe-Masselot, Christel Neut, Elisabeth Singer, Ségolène Plet, Karel Geboes, Pierre Desreumaux, Frédéric Gottrand and Jean-Luc Desseyn

Supplementary Information

Supplemental Table 1: Oligonucleotides used for TaqMan RT–qPCR.

Supplemental Table 2. Histological damage and inflammation after DSS-treatment.

Supplemental Figure 1: Profile expression of the transgene product. a. Visualization by western blotting of the secretion of the transgenic product into the colonic mucus using an anti-CYS domain antibody. **b.** Profile expression of the transgene product using an anti-GFP antibody. Cells were counterstained with Hoechst 33258 (blue). Lu, lumen. **c.** Immunofluorescence of fresh opened colon (green fluorescent protein (GFP) is depicted in green).

Supplemental Figure 2: RT–qPCR analysis of mucin expression. a. Muc2 and Muc6 expression was measured by RT–qPCR (TaqMan) in triplicate using colonic cDNA from wild-type (WT; n = 8) and transgenic (Tg, n = 12) mice. **b.** Muc1, Muc3 and Muc4 expression was measured by RT–qPCR (TaqMan) in triplicate using colonic cDNA from wild-type (WT; n = 5) and transgenic (Tg, n = 6) mice. ns, nonsignificant.

Supplemental Figure 3: The gut barrier epithelium of Tg mice is not modified. a. Immunofluorescence images of colon stained for the claudin-7 and occludin (green)

transmembrane tight-junction proteins showed that transgenic (Tg) mice did not have alterations in epithelial tight junctions. Cells were counterstained with Hoechst 33258 (blue). **b.** The intestinal epithelial permeability to FITC-dextran was similar between wild-type (WT) and Tg mice. ns, nonsignificant.

Supplemental Figure 4: Modification of colonic mucin glycosylation. a. Colonic sections showing a preserved mucus in transgenic (Tg) mice, which harbored more sialic acid α-2,3-galactose epitopes, as visualized using the MAA lectin (in red). Muc2 is visualized in green. Cells were counterstained with Hoechst 33258 (blue). **b.** Nano-electrospray-mass spectrometry of oligosaccharides from wild-type (WT) and Tg colonic mucins acquired in the negative ion mode. The oligosaccharide compositions of major peaks are indicated as 5 numbers separated by comma for Hex, HexNAc, Fuc, NeuAc, and SO_3, respectively.

Supplemental Video 1: Intrarectal endoconfocal imaging of anesthetized mice. Left, wild-type (WT) mouse; middle and right, transgenic (Tg) mouse (colorectal and colon, respectively).

Supplemental Video 2: Endoconfocal imaging of the gallbladder of a transgenic mouse.

Supplemental Table 1. Oligonucleotides used for TaqMan RT-qPCR

Gene	Sequences (5' to 3')*	Product length
Tnfalfa	FW TCTCAAAATTCGAGTGACAAGC	
	RV CAGCCACTCCAGCTGCTC	
	PB GCCCACGTCGTAGCAAACCACC	75bp
IL1beta	FW TGTGAAATGCCACCTTTTGA	
	RV CAGGTCAAAGGTTTGGAAGC	
	PB CGGACCCCAAAAGATGAAGGGC	96bp
IL6	FW GTTCTCTGGGAAATCGTGGA	
	RV TTCTGCAAGTGCATCATCGT	
	PB GAGTTGGCAATGGCAATTCTGATTG	84bp
IL17alpha	FW CTCAACCGTTCCACGTCAC	
	RV TGAGCTTCCCAGATCACAGA	
	PB TCCACCGCAATGAAGACCCTGA	80bp
IFNgamma	FW TTTGAGGTCAACAACCCACA	
	RV ATCAGCAGCGACTCCTTTTC	
	PB TGCCGGAATCCAGCCTCAGG	115bp
Muc2	FW CGACCTGAGAACTGGAGGAC	
	RV CCAGATGTGAGCATGTGTCG	
	PB TCTGCCCCAAGAAATGCCCC	105bp
Muc1	FW TGAGTGAATACCCTACCTACCACA	
	RV GCTGGGTTGTAAGAGAGACTG	
	PB CCCCTATGAGGAGGTTTCGGCAGGTAA	123bp
Muc3/Muc17	FW GACTCTGTGTACAACACCTTCCAG	
	RV AAACCGCTTTTGTGTTAGTGTTT	
	PB AAAGAAAGATCCAGATTCAGAGGCCCC	120bp

*FW, forward; RV, reverse; PB, probe.

Supplemental Table 2. Histological damage and inflammation after DSS-treatment

feature graded	wild-type	transgenic	*P*-value
severity	1.45 ± 0.82	0.67 ± 1.00	0.022
extent	1.18 ± 0.75	0.56 ± 0.72	0.056
regeneration	1.09 ± 0.94	0.22 ± 0.67	0.025
crypt damage	0.64 ± 0.80	0.22 ± 0.66	ns
percent involvement	1.64 ± 0.67	1.22 ± 0.67	0.058

Histological grading of colitis in wild-type (n=11) and transgenic mice (n=9). Data are means ± SE. ns, nonsignificant.

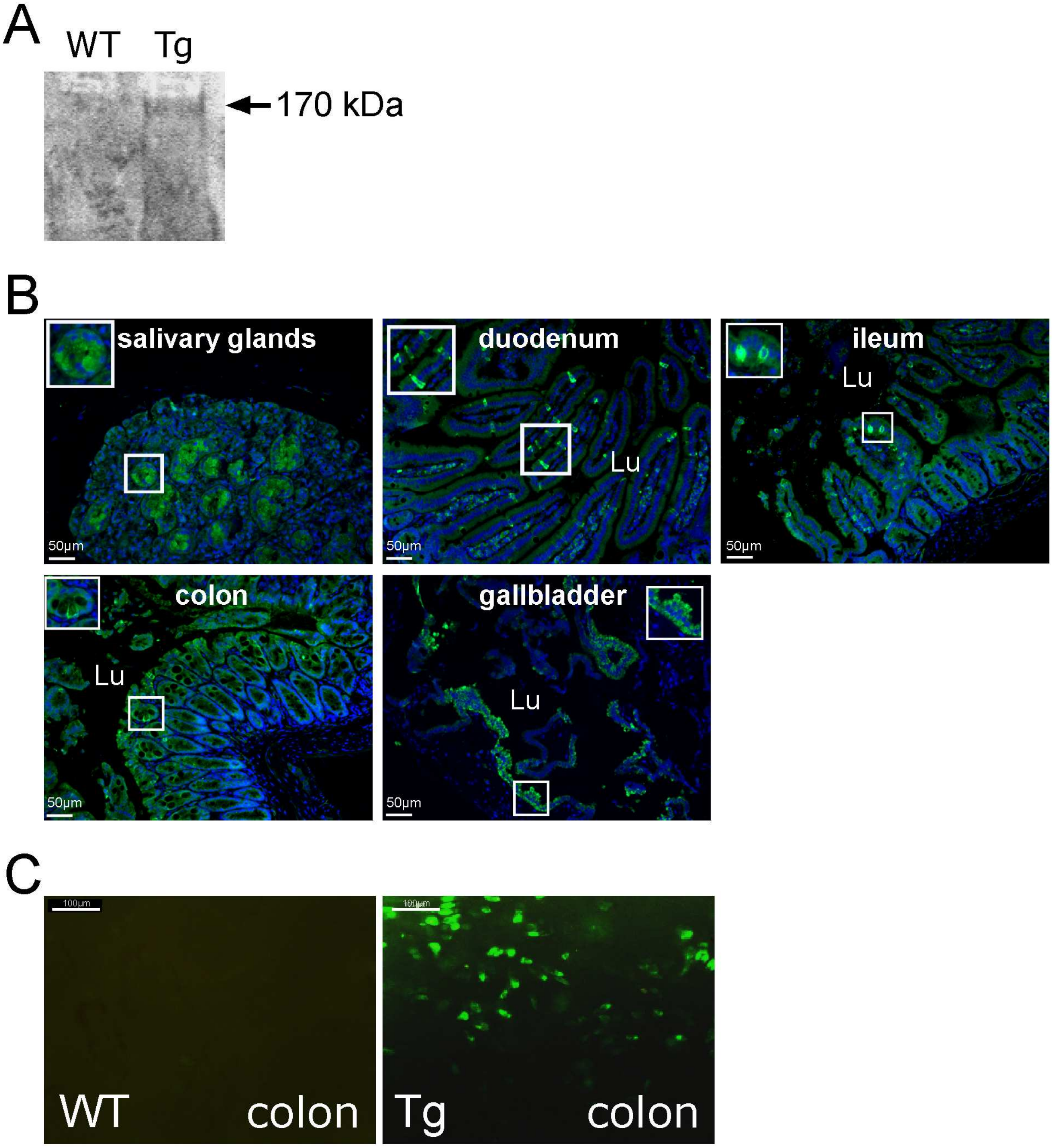
A
WT
Tg
170 kDa
B
salivary glands
duodenum
ileum
colon
gallbladder
Lu
50µm
C
100µm
WT
colon
Tg
colon

Suppl. Fig. 1

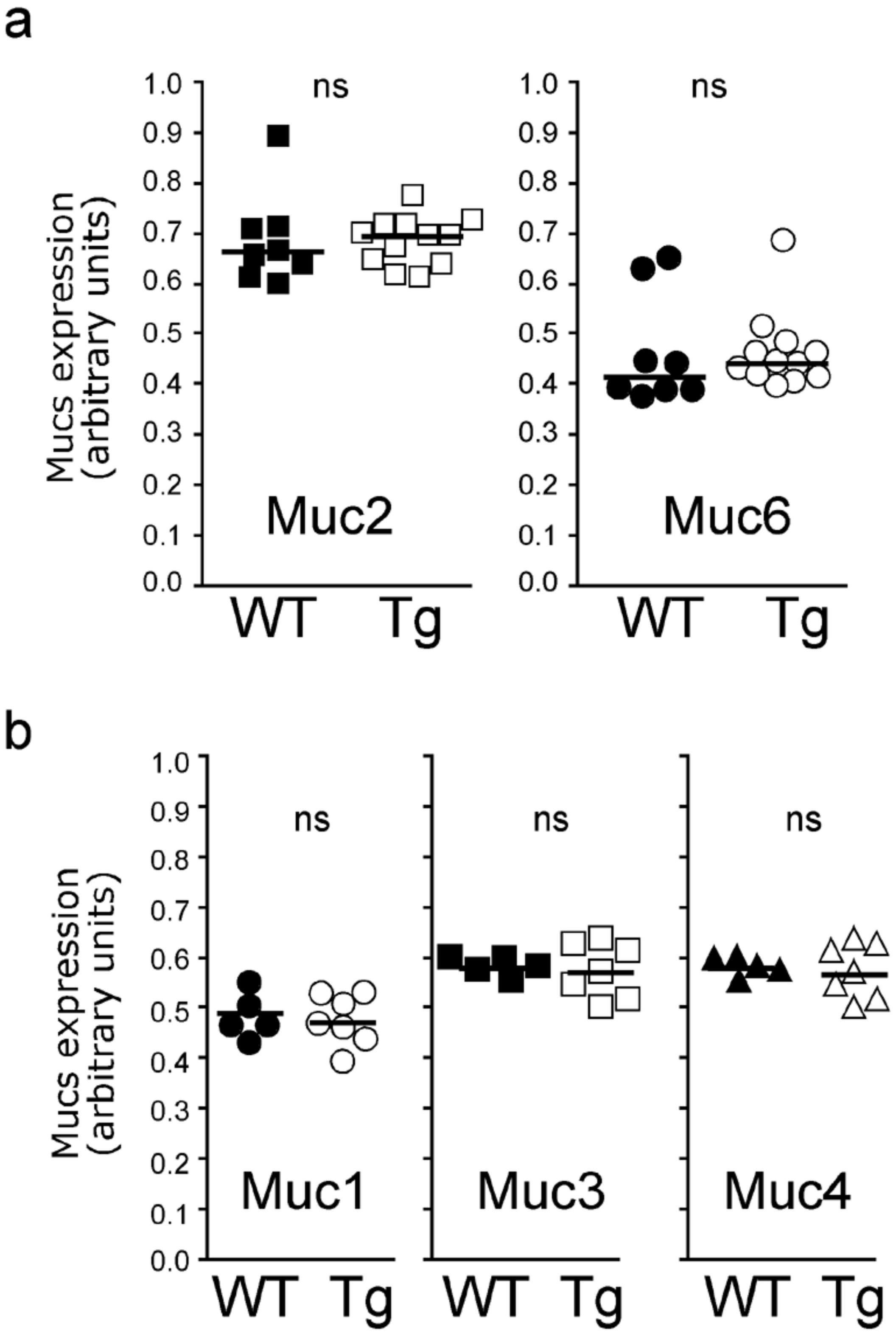

Suppl. Figure 2

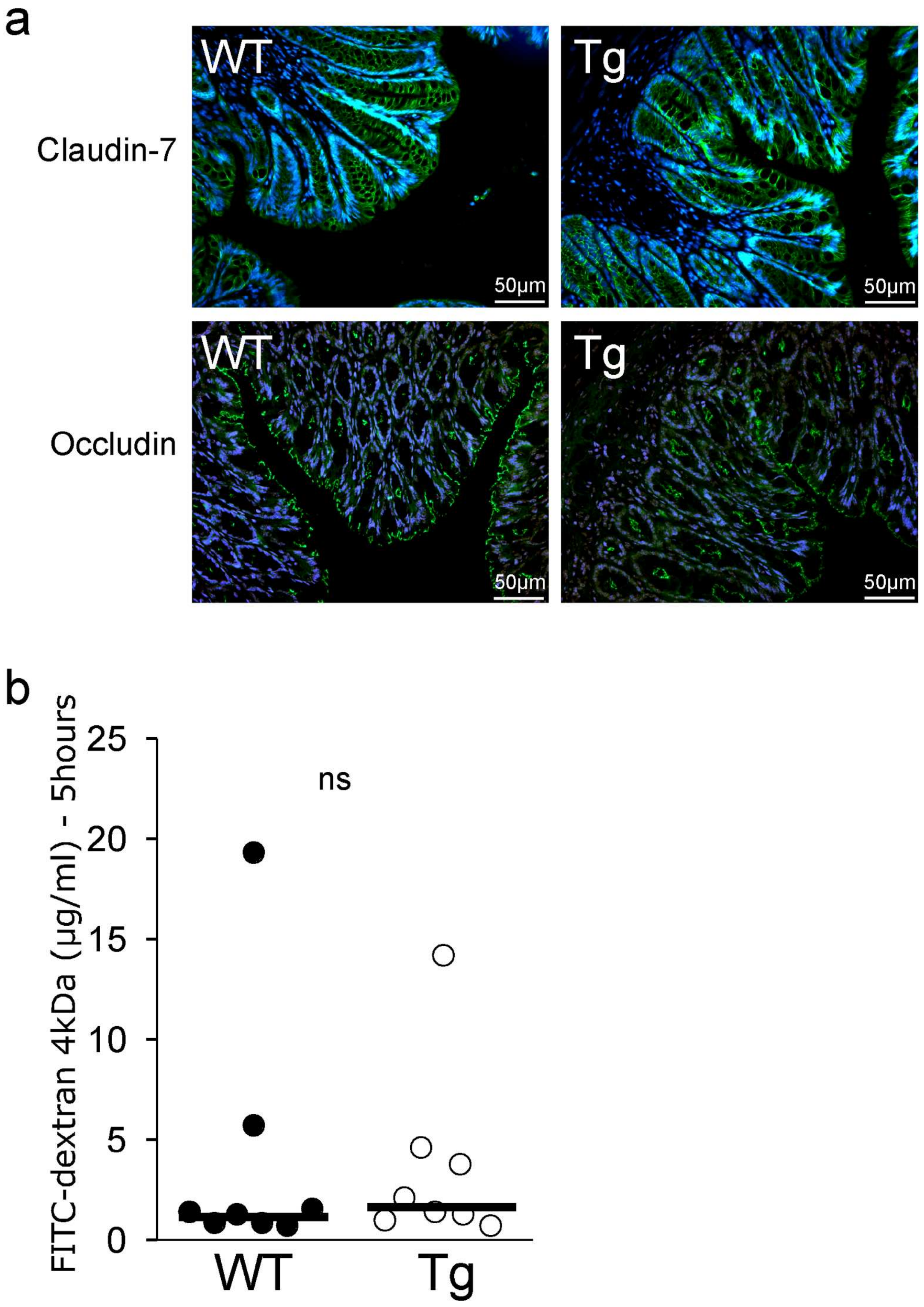
a
WT
Tg
Claudin-7
50μm
50μm
WT
Tg
Occludin
50μm
50μm
b
FITC-dextran 4kDa (μg/ml) - 5hours
25
20
15
10
5
0
ns
WT
Tg

Suppl. Fig. 3

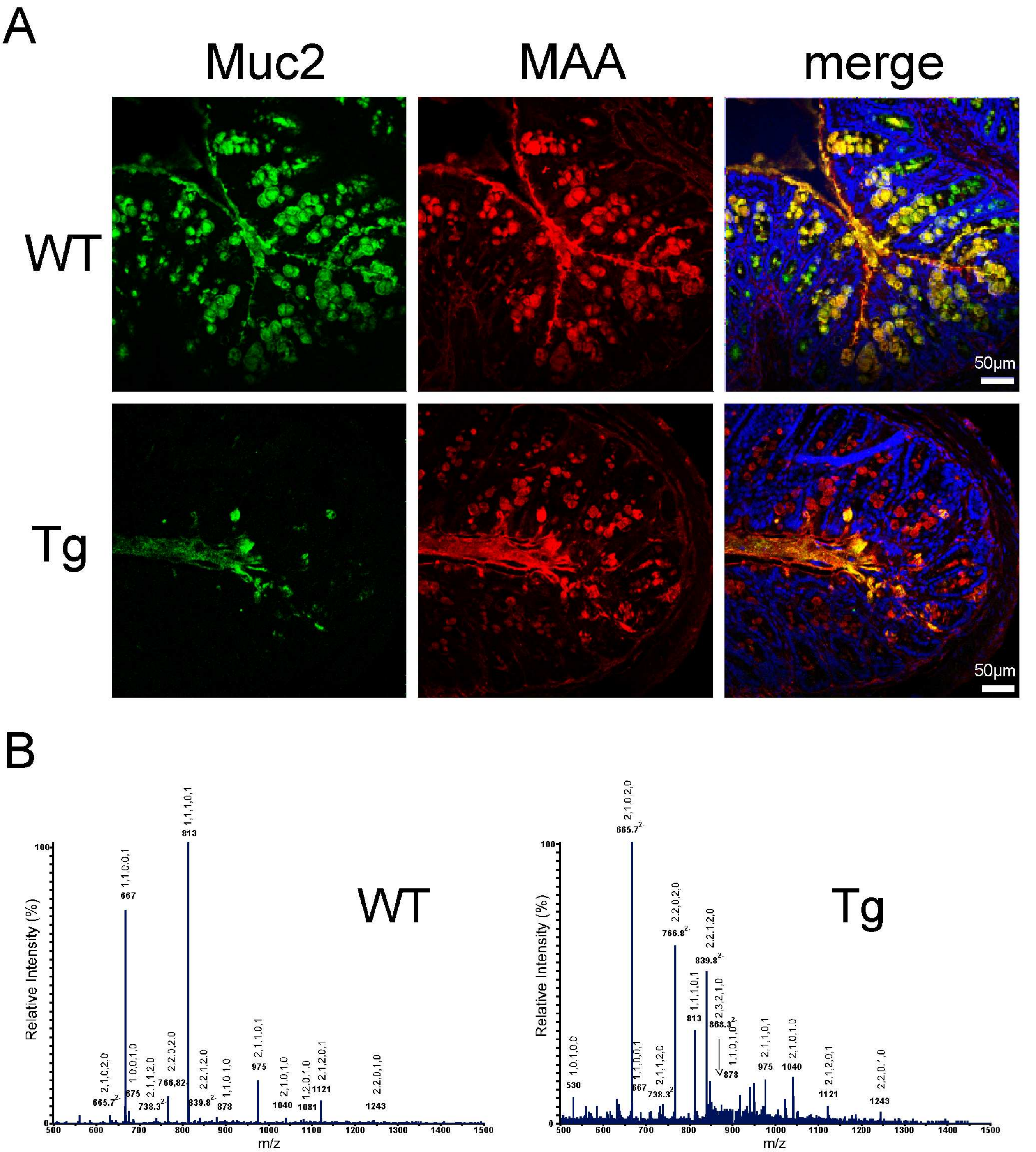

Suppl. Fig. 4
